# Differential Effects of Source-Specific Particulate Matter on Emergency Hospitalizations for Ischemic Heart Disease in Hong Kong

**DOI:** 10.1289/ehp.1307213

**Published:** 2014-02-07

**Authors:** Vivian Chit Pun, Ignatius Tak-sun Yu, Kin-fai Ho, Hong Qiu, Zhiwei Sun, Linwei Tian

**Affiliations:** 1 The Jockey Club School of Public Health and Primary Care, The Chinese University of Hong Kong, Hong Kong Special Administrative Region of the People’s Republic of China; 2 School of Public Health and Family Medicine, Capital Medical University, Beijing, People’s Republic of China

## Abstract

Background: Ischemic heart disease (IHD) is a major public health concern. Although many epidemiologic studies have reported evidence of adverse effects of particulate matter (PM) mass on IHD, significant knowledge gaps remain regarding the potential impacts of different PM sources. Much the same as PM size, PM sources may influence toxicological characteristics.

Objectives: We identified contributing sources to PM_10_ mass and estimated the acute effects of PM_10_ sources on daily emergency IHD hospitalizations in Hong Kong.

Methods: We analyzed the concentration data of 19 PM_10_ chemical components measured between 2001 and 2007 by positive matrix factorization to apportion PM_10_ mass, and used generalized additive models to estimate associations of interquartile range (IQR) increases in PM_10_ exposures with IHD hospitalization for different lag periods (up to 5 days), adjusted for potential confounders.

Results: We identified 8 PM_10_ sources: vehicle exhaust, soil/road dust, regional combustion, residual oil, fresh sea salt, aged sea salt, secondary nitrate, and secondary sulfate. Vehicle exhaust, secondary nitrate, and secondary sulfate contributed more than half of the PM_10_ mass. Although associations with IQR increases in 2-day moving averages (lag_01_) were statistically significant for most sources based on single-source models, only PM_10_ from vehicle exhaust [1.87% (95% CI: 0.66, 3.10); IQR = 4.9 μg/m^3^], secondary nitrate [2.28% (95% CI: 1.15, 3.42); IQR = 8.6 μg/m^3^], and aged sea salt [1.19% (95% CI: 0.04, 2.36); IQR = 5.9 μg/m^3^] were significantly associated with IHD hospitalizations in the multisource model. Analysis using chemical components provided similar findings.

Conclusion: Emergency IHD hospitalization was significantly linked with PM_10_ from vehicle exhaust, nitrate-rich secondary PM, and sea salt–related PM. Findings may help prioritize toxicological research and guide future monitoring and emission-control polices.

Citation: Pun VC, Yu IT, Ho KF, Qiu H, Sun Z, Tian L. 2014. Differential effects of source-specific particulate matter on emergency hospitalizations for ischemic heart disease in Hong Kong. Environ Health Perspect 122:391–396; http://dx.doi.org/10.1289/ehp.1307213

## Introduction

Over the past decades, epidemiologic evidence has linked ambient particulate matter (PM) pollution to increased cardiovascular morbidity and mortality (Dominici et al. 2006; Peng et al. 2009). Of the cardiovascular end points, ischemic heart disease (IHD) is a major public health concern. IHD is defined as a narrowing of the coronary vessels that supply blood to the heart muscle. It was the leading cause of death worldwide in 2008 and the second leading cause of death in 2011 in Hong Kong, which had a population of around 7 million and a daily average of 12 IHD deaths that year (Department of Health HKSAR 2013). Evidence from the United States and Europe of increases in IHD events after acute exposure to elevated PM concentrations has been convincing (Dominici et al. 2006; Forastiere et al. 2005; Pope et al. 2006). Dominici et al. (2006) estimated that an average 10-μg/m^3^ reduction in PM_2.5_ (≤ 2.5 μm in aerodynamic diameter) in 204 U.S. counties would prevent > 1,500 IHD hospitalizations per year. However, previous studies in Hong Kong did not observe associations between PM and IHD hospitalizations (Wong CM et al. 2002; Wong TW et al. 1999) or mortality upon adjusting for gaseous pollutants (Wong TW et al. 2002). Heterogeneity in findings may reflect the fact that PM is a complex mixture of particles that vary in physical attributes, chemical composition, solubility, and emission sources (Pope and Dockery 2006).

Growing research emphasis has been placed on PM sources and chemical composition (Health Effects Institute 2002; National Research Council 2004). Because PM sources generate mixtures of air pollutants with different physicochemical compositions, the source might affect the relative toxicity of PM. This hypothesis is supported by toxicological evidence suggesting that PM-induced biologic effects can depend on the zone of origin (e.g., the industrial zone; Alfaro-Moreno et al. 2002). Currently, the majority of studies have associated IHD, especially myocardial infarction, with traffic-related pollution exposures estimated using surrogate pollutants (e.g., PM_2.5_ mass, carbon monoxide, nitrogen dioxide) or direct-exposure data (e.g., time spent in traffic) (D’Ippoliti et al. 2003; Lanki et al. 2006b; Peters et al. 2004). Despite these findings, it has been a challenge to quantitatively assess the impacts of multiple PM emission sources on IHD. Associations with PM sources have been inconsistent across existing studies. Although some studies have reported associations of traffic-related and/or combustion-generated PM with increases in repolarization, inflammatory markers, and ST segment depressions among IHD patients (Lanki et al. 2006a; Yue et al. 2007), others have reported that IHD hospitalizations were not linked with traffic-related particles or other PM sources (Halonen et al. 2009; Lall et al. 2011).

In Hong Kong, although research on PM pollution and health outcomes has been active since the late 1990s, specific PM chemical components and sources responsible for the adverse effects have rarely been investigated. In the present study, we took advantage of the PM_10_ (≤ 10 μm in aerodynamic diameter) speciation data that have been available for over a decade to identify contributing sources to PM_10_ mass using a source apportionment model and then used those data to estimate the acute effects of PM_10_ sources on daily emergency IHD hospital admissions.

## Methods

*Data*. The Hong Kong Environmental Protection Department has been collecting 24-hr filter samples of PM_10_ regularly at six general and one roadside air quality monitoring stations since 2001 (Yuan et al. 2013). These monitoring stations were interspersed in different districts of Hong Kong. We included only data from the six general stations that are not in direct proximity to traffic, industrial sources, buildings, or residential sources of emissions from the burning of coal, waste, or oil. These stations serve to capture the air quality that the general population is exposed to on a regular basis. Twenty-six PM_10_ chemical components were speciated from the filter samples via various analytical methods as described in detail previously (Yuan et al. 2013). We included speciation data from between 1 January 2001 and 31 December 2007 in the present study. The PM_10_ sampling frequency was on average every 6th day, with each station operated on a distinct sampling schedule. On a particular day, there might be no or multiple samples taken across the stations. Overall, 71% of the study days were covered by measurements from at least one station. We obtained daily mean temperature and relative humidity from the Hong Kong Observatory for the same study period.

We acquired daily counts of emergency hospital admissions for between 1 January 2001 and 31 December 2007 from the Hong Kong Hospital Authority (Wong TW et al. 1999). Data were coded according to the *International Classification of Diseases, 9th Revison* (ICD-9; World Health Organization 1977). Hospitalizations for IHD (ICD-9 codes 410–414) were extracted to construct the time series. Hospitalizations due to influenza (ICD-9 code 487) were extracted and treated as a potential confounder in the regression analysis.

*Statistical analysis*. We first used the U.S. Environmental Protection Agency’s (EPA) Positive Matrix Factorization (PMF), version 3.0 (http://www.epa.gov/heasd/research/pmf.html), to identify a set of factors interpreted as emission sources and to estimate the source contributions to PM_10_ mass (Hopke 2008). Station-specific measurements of elemental carbon (EC), organic matter (OM), nitrate (NO_3_^–^), sulfate (SO_4_^2–^), ammonium ion (NH_4_^+^), chloride ion (Cl^–^), sodium ion (Na^+^), potassium ion (K^+^), aluminum (Al), arsenic (As), calcium (Ca), cadmium (Cd), iron (Fe), magnesium (Mg), manganese (Mn), nickel (Ni), lead (Pb), vanadium (V), and zinc (Zn) of PM_10_ were entered into the PMF model. Details on PMF modeling have been described previously (Reff et al. 2007).

We removed the station-specific influence on the resultant concentrations of each PM_10_ source by *a*) computing the annual mean concentration (*X_i_*) for each monitoring station *i*, *b*) subtracting the annual mean from the daily mean concentration for station *i* on each sample day *j* (*X_ij_*), *c*) adding the annual mean of all stations (*X*) to the resulting centered values (*X_ij_* – *X_i_*) for each station and sampling day to produce *X´´_ij_* = *X_ij_* – *X_i_* + *X*, and *d*) taking the average of *X´´_ij_* over all stations (Wong CM et al. 2001). The final PM_10_ sources time series contained nonmissing territorywide mean concentrations of PM_10_ sources for 1,805 days (71% of the 2,556 total days), which is about 5 days/week. All pollutant concentrations were expressed inmicrograms per meter cubed, except for EC and OM, which were reported in microgram of carbon per meter cubed.

Generalized additive models with log link and Poisson error were used to estimate the associations between PM_10_ sources and emergency IHD hospital admissions (Hastie and Tibshirani 1990). We adopted *a priori* model specification to guide the selection of degrees of freedom (df) for time-varying variables: smoothing splines with 8 df per year for time trend, 6 df for current day temperature and previous 3-days moving average, and 3 df for current day relative humidity and previous 3-days moving average (Bell et al. 2009; Peng et al. 2009). We included dummy variables for day of week, public holidays, and influenza epidemics (Wong CM et al. 2002).

We investigated the possible lag distribution of associations with each PM_10_ source for exposures on the same day (lag_0_) and for daily exposures on the previous 1–5 days (lag_1_ to lag_5_). However, we focused primarily on the 2-day moving average of exposure on the same day and the previous day (lag_01_) *a priori* based on previous studies (Wong CM et al. 2002, 2008). Furthermore, we conducted multisource analyses to estimate mutually adjusted effects of PM_10_ sources on emergency IHD hospitalizations (Ostro et al. 2011). To minimize multicollinearity, we used backward elimination with an exclusion criterion of *p* > 0.10 to select PM sources to include in the final multisource model while controlling for time trend, seasonality, meteorological conditions, calendar effects, and influenza epidemics. Pearson’s correlations were used to summarize the relationships between source-apportioned PM_10_. PM_10_ “tracer” components, which are characterized as the typical components that are exclusively or largely derived from a particular source, were also examined, and those tracers that are found specifically in the sources included in the final multisource model were further tested in a separate multipollutant model to validate the multisource findings. A smoothing function with 3 df was applied to graphically describe the relationships between sources and IHD hospitalizations while adjusting for time-varying confounders. For sensitivity analyses, we repeated the time-series analyses after either imputing source concentrations for the days without samples from any stations (751 days) by linear interpolation using the na.approx function in the R zoo package or by replacing the missing data with nonmissing measurement values from the previous day. Moreover, we evaluated the impact of alternative df values (5–12) for time trend on the risk estimates. All estimates were reported as the percent increase [(relative risk – 1) × 100%] in daily emergency IHD hospital admissions for an interquartile range (IQR) increment in pollutant concentrations. Where appropriate, 95% confidence intervals (CIs) were calculated. We performed all time-series analyses in the statistical environment R Software, version 2.15.0 (R Foundation for Statistical Computing, Vienna, Austria).

## Results

**Figure 1:**
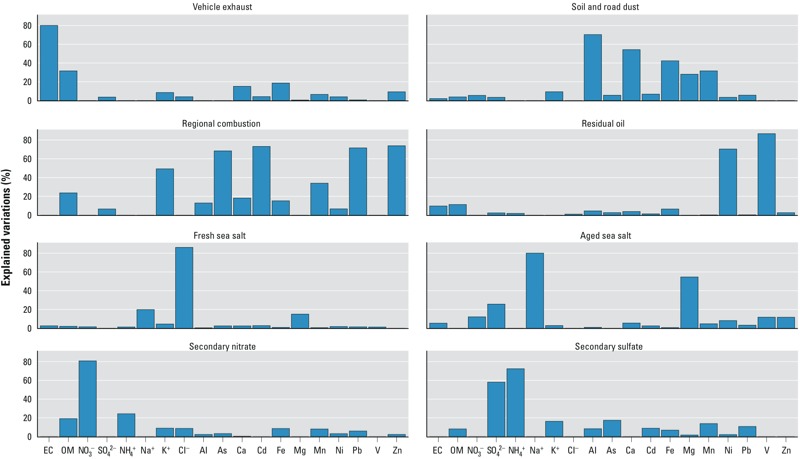
PM_10_ source profiles, indicated by explained variations that estimate how much a source explains the variation of a particular chemical component.

We identified 8 PM_10_ sources, namely vehicle exhaust, soil/road dust (e.g., from exposed soil, unpaved roads), regional combustion, residual oil combustion (e.g., fuel emissions from marine vessels), fresh sea salt, aged sea salt, secondary nitrate, and secondary sulfate. Figure 1 shows the estimated PM_10_ source profiles, depicted as explained variations that indicate the relative contribution of each source to the variation of a given chemical component (Paatero and Tapper 1994). For instance, vehicle exhaust emission accounted for 80% of the variation in EC. Regional combustion emission was identified as a composite of two sources that could not be further separated. They were wood/biomass burning [based upon the abundance (i.e., a large explained variation) of K^+^] and coal combustion in power plants and industrial facilities in the adjacent Pearl River Delta region (based upon the abundances of As, Cd, Pb, and Zn in the source profile, which cannot be further separated) (Yuan et al. 2013). Table 1 summarizes the levels of PM_10_ pollution, weather conditions, and IHD hospital admission counts. Between 2001 and 2007, the daily average concentration for PM_10_ in Hong Kong was 55.8 ± 32.5 μg/m^3^. Secondary sulfate accounted for the largest fraction of total PM_10_ mass (23.6%), followed by vehicle exhaust (15.1%) and secondary nitrate (14.9%). The mean daily average temperature and relative humidity were 23.6°C and 78.3%, respectively (Table 1). During the study period, there were 76,659 hospitalizations for IHD (30 ± 7 admissions per day).

**Table 1:** Descriptive statistics for PM_10_ sources, meteorological factors, and number of emergency hospital admissions in Hong Kong, 2001–2007.

Variable	No. of days	Daily mean ± SD	Percent of PM_10_	IQR
Emergency hospital admissions (counts)
IHD	2,556	30 ± 7		9.0
Meteorological conditions
Temperature (°C)	2,556	23.6 ± 4.9		8.1
Relative humidity (%)	2,556	78.3 ± 9.9		11.4
PM_10_ concentration (μg/m^3^)
Total PM_10_	1,805	55.8 ± 32.5	100.0	44.8
Vehicle exhaust	1,805	8.4 ± 3.7	15.1	4.9
Soil/road dust	1,805	7.5 ± 9.0	13.4	6.9
Regional combustion	1,805	7.5 ± 9.3	13.5	11.7
Residual oil	1,805	2.4 ± 2.5	4.3	2.2
Fresh sea salt	1,805	2.1 ± 2.7	3.7	2.0
Aged sea salt	1,805	7.2 ± 4.4	12.8	5.9
Secondary nitrate	1,805	8.3 ± 8.8	14.9	8.6
Secondary sulfate	1,805	13.2 ± 12.7	23.6	15.8

**Figure 2:**
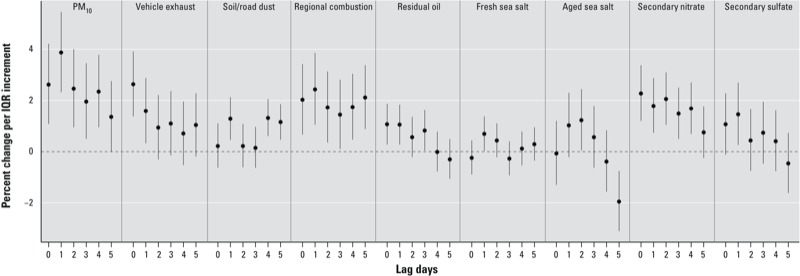
Percent change (95% CI) in emergency IHD hospital admissions per IQR increment in PM_10_ mass and sources at different lag periods, adjusted for meteorological factors, seasonal and temporal trend, day of week, and influenza epidemics. See Table 1 for individual IQR values.

**Figure 3:**
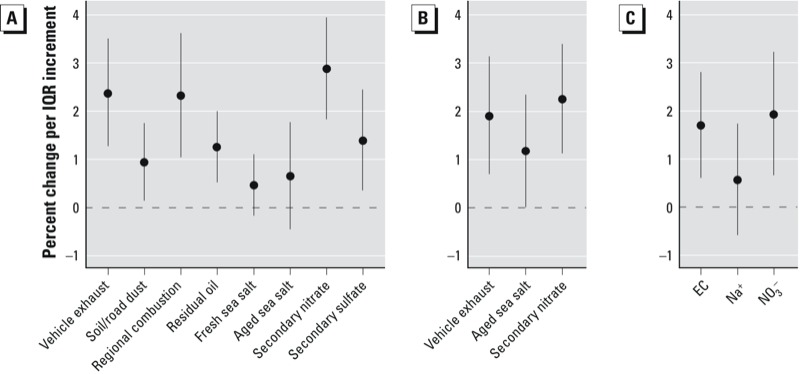
Percent change (95% CI) in emergency IHD hospital admissions per IQR increment in 2-day moving average concentration (lag_01_) of PM_10_ sources based on single-source models (*A*), a multisource model (*B*), and PM_10_ trace elements based on multipollutant model (*C*). All models were adjusted for time trend, seasonality, meteorological conditions, calendar effects, and influenza epidemics. See Table 1 for individual IQR values for sources, and the IQR for EC (tracer for vehicle exhaust), Na^+^ (for aged sea salt), and NO3^–^ (for secondary nitrate) was 1.6, 1.2, and 3.4 μg/m^3^, respectively.

Single-source models of single-day exposure lags showed similar patterns of associations for most of the PM_10_ sources, in that IHD hospitalizations were positively associated with exposure on the same day (lag_0_), maximal for lag_0_ or lag_1_, and lowest at later lags (lag_4_–lag_5_) (Figure 2). At lag_01_ (Figure 3A), the source that was most strongly associated with IHD hospitalizations was secondary nitrate [2.89% increase (95% CI: 1.83, 3.95); IQR = 8.6 μg/m^3^], followed by vehicle exhaust [2.35% (95% CI: 1.24, 3.47); IQR = 4.9 μg/m^3^] and regional combustion [2.26% (95% CI: 0.98, 3.55); IQR = 11.7 μg/m^3^], after adjusting for time trend, seasonality, meteorological conditions, calendar effect, and influenza epidemics. Significant positive associations were also found for particles originated from soil/road dust (per 6.9 μg/m^3^), residual oil (per 2.2 μg/m^3^), and secondary sulfate (per 15.8 μg/m^3^), corresponding to estimated increases in IHD hospitalizations of 0.97–1.37%.

Correlations between source-apportioned PM_10_ were nil to moderate. The highest correlation coefficient, 0.67, was between regional combustion and secondary sulfate, followed by 0.59, between regional combustion and secondary nitrate (Table 2). Backward elimination resulted in a multisource regression model that included vehicle exhaust, aged sea salt, and secondary nitrate sources only (Figure 3B). All other sources, although statistically significant in single-source models, were eliminated from the final multisource model on the basis of *p* > 0.10. The estimated effects of vehicle exhaust [1.87% (95% CI: 0.66, 3.10)] and secondary nitrate [2.28% (95% CI: 1.15, 3.42)] at lag_01_ were slightly attenuated relative to single-source model estimates but remained significant in the final multisource model. The association between aged sea salt and IHD hospitalizations was stronger based on the multisource model [1.19% (95% CI: 0.04, 2.36); IQR = 5.9 μg/m^3^] compared with the single-source model [0.68% (95% CI: –0.43, 1.79)]. Associations with tracer components of secondary nitrate and vehicle exhaust were similar, that is, NO_3_^–^ (tracer for secondary nitrate) and EC (tracer for vehicle exhaust) were associated with 1.95% [(95% CI: 0.68, 3.25); IQR = 3.4 μg/m^3^] and 1.67% [(95% CI: 0.58, 2.78); IQR = 1.6 μg/m^3^] increases in IHD hospitalizations, respectively (Figure 3C).

**Table 2:** Pearson’s correlation among the estimated sources of PM_10_.

	PM_10_	Vehicle exhaust	Soil/road dust	Regional combustion	Residual oil	Fresh sea salt	Aged sea salt	Secondary nitrate
PM_10_	1.0
Vehicle exhaust	0.48	1.00
Soil/road dust	0.58	0.21	1.00
Regional combustion	0.84	0.49	0.38	1.00
Residual oil	0.40	0.35	–0.02	0.29	1.00
Fresh sea salt	0.10	–0.18	0.12	–0.07	–0.11	1.00
Aged sea salt	0.07	–0.27	0.08	–0.22	–0.11	0.23	1.00
Secondary nitrate	0.76	0.34	0.29	0.59	0.34	0.24	0.05	1.00
Secondary sulfate	0.78	0.30	0.20	0.67	0.38	–0.15	–0.02	0.43

We examined the concentration–response relations for vehicle exhaust, aged sea salt, and secondary nitrate in a multisource model. We observed moderate positive relationships over the IQRs of source concentrations, except for aged sea salt, where a neutral relationship was seen (data not shown). The risk estimates were not sensitive to alternative time-series models in which we imputed missing data (data not shown). Varying df for time trend (5–12 per year, data not shown) did not substantially change the regression results either.

## Discussion

Research directly delineating the health impacts of PM emission sources is relatively limited. Most studies rely on ambient concentrations of a PM chemical component as a surrogate of the combined exposure to one source (Stanek et al. 2011). This *a priori* selection can be complicated when interpreting the results because many components are emitted from numerous sources and the same component may not serve as tracer to the same source at different locations (Sarnat et al. 2008). We joined a small but growing number of epidemiologic studies to conduct source apportionment analysis and quantitatively estimate the associations between multiple PM sources and health outcome. Early short-term air pollution studies conducted in western countries identified some associations between PM sources and mortality (Cakmak et al. 2009b; Halonen et al. 2009; Ito et al. 2006; Laden et al. 2000; Mar et al. 2000, 2006; Ostro et al. 2011), and gradually, researchers have also linked certain PM sources to hospital admissions (Andersen et al. 2007; Cakmak et al. 2009a; Halonen et al. 2009; Lall et al. 2011; Sarnat et al. 2008). Overall, these studies have reported some evidence suggesting that PM sources representing traffic/motor exhausts, regional/secondary sulfate, and coal/oil combustion may be more toxic than other PM sources, as summarized in a recent review by Stanek et al. (2011). Nonetheless, there is insufficient evidence to draw more specific conclusions across studies. Because emission sources of air pollutants vary not only temporally but also geographically, studies on source-apportioned PM mass under different atmospheres are needed to improve our understanding of PM-related health effects.

To our knowledge, this is the first Asian study to investigate the health impacts of multiple PM sources. We estimated the associations of short-term exposure to source-apportioned PM_10_ mass with emergency IHD hospital admissions in Hong Kong, a coastal urban city on the boundary region of Asian continent and Pacific Ocean. In contrast to previous studies conducted in New York City, New York (USA), and Helsinki, Finland, that reported no associations of PM_2.5_ sources with IHD hospital admissions (Halonen et al. 2009; Lall et al. 2011), we observed significant associations between IQR increases in several PM_10_ sources and daily IHD hospitalizations for single-day lag periods up to 5 days prior. Differences between our findings and those of previous studies might be related to the longer study period (7 years), larger combined sample size in the present study, as well as the differences in pollution compositions and population susceptibility between cities. Although precise pathophysiological mechanisms connecting ambient air pollution with IHD remain to be determined, it is commonly hypothesized that PM sources may trigger and/or enhance the formation of reactive oxygen species that induce inflammation, the formation of atherosclerotic plaques, and vasoconstriction, resulting in reduced oxygen supply of heart tissues, and thereby leading to IHD (Lawal and Araujo 2012; Peters 2011).

Secondary nitrate in PM_10_ (per 8.6 μg/m^3^) was associated with the largest increases in IHD hospitalizations at lag_01_ in both single-source and multisource models. However, the estimated association of secondary sulfate diminished after adjusting for other sources, and secondary sulfate was dropped from the final multisource model based on the backward elimination criterion (data not shown). This was somewhat surprising considering that secondary sulfate accounted for the largest fraction of total PM_10_ in Hong Kong. Whereas most studies that examined these associations found that sulfate-rich secondary PM was more strongly associated with mortality and hospital admissions than nitrate (e.g., Ito et al. 2006; Mar et al. 2000, 2006; Sarnat et al. 2008), a few studies have reported that nitrate in PM_2.5_, rather than sulfate, was significant predictor of mortality (Fairley 1999; Ostro et al. 2011). Secondary nitrate and secondary sulfate, respectively, derive largely from the oxidation of nitrogen oxides and sulfur dioxide emitted from combustion of fossil fuels. Although both are acidic in nature, their strength of acidity varies greatly depending upon the city-specific interactions between local emissions, regional transports, and meteorological conditions (Schlesinger and Cassee 2003). Although animal toxicological evidence is inconclusive, Kelly and Fussell (2012) hypothesized that acidic aerosols may lower the pH within the airways by depositing hydrogen ions, thereby triggering adverse reactions. In China, strong economic growth and high total energy consumption have led to substantial increases in anthropogenic nitrogen and sulfur emissions over the past decades (Liu et al. 2013; Lu et al. 2010). Studies showed that emissions of nitrogen oxides and sulfur dioxide in the adjacent Pearl River Delta region due to rapid industrialization and urbanization have been the dominant contributors to secondary nitrate and secondary sulfate in Hong Kong through regional transportation (Guo et al. 2009; Yuan et al. 2013). Our finding on secondary nitrate is of particular importance for lending urgency to policy makers, particularly in developing economies, regarding both local and regional emission control and reduction of gaseous pollutants.

We found that an IQR increment (4.9 μg/m^3^) in the 2-day moving average concentration of PM_10_ from vehicle exhaust was associated with a 1.91% estimated increase (95% CI: 0.70, 3.13%) in IHD hospital admissions after adjusting for other statistically significant sources. In accordance with these results, EC as a chemical tracer of vehicle exhaust (largely from diesel engines) was also significantly associated with IHD hospitalization risk. Vehicle exhaust-related PM refers to combustion-derived particles that primarily accumulate in the fine fraction of PM_10_. Previous epidemiologic studies have reported that mobile sources PM_2.5_ and EC are stronger predictors of overall cardiovascular outcomes than other sources and components (Cakmak et al. 2009a, 2009b; Lall et al. 2011; Mar et al. 2006; Ostro et al. 2011; Sarnat et al. 2008), which is consistent with our finding of an association of IHD with vehicle exhaust PM. Plausible biological mechanisms include elevated levels of inflammatory biomarkers, impaired endothelium-dependent vasodilation, and promotion of ST-segment depression (Dales et al. 2007; Lanki et al. 2006a; Yue et al. 2007). In Hong Kong, where road density was among the highest in the world at 254 vehicles per kilometer of road in 2009, exposure to traffic-related air pollution is ubiquitous (World Bank 2012). These findings on vehicle exhaust particles stress the importance of the continuous reduction of overall traffic and related emissions and the reconfiguration of urban environments to reduce personal exposure to traffic.

We observed that aged sea salt was associated with an increased risk of IHD hospitalizations after adjusting for other sources. Sea salts are most abundantly found in the coarser fraction of PM_10_. Whereas Mar et al. (2006) reported that sea salt was consistently associated with elevated cardiovascular and total mortality in Atlanta, Georgia (USA), across the various source-apportionment analyses in an inter-method comparison study, most epidemiologic studies that estimated this association found no relationship between sea salt and health outcomes (Andersen et al. 2007; Gent et al. 2009; Lanki et al. 2006a; Ostro et al. 2011). Lee et al (2008) and Zhuang et al. (1999) suggested possible relations between sea salt and secondary nitrate because nitric acid may react with marine particles to form coarse mode nitrate along coastal areas. However, we observed null-to-weak correlations between secondary nitrate and sea salts. This association should be investigated further.

Our findings add to the existing literature in several ways. First, we examined air pollution association with a specific cardiovascular end point, as opposed to a broad composite end point of different cardiovascular events, to provide better insight into the plausible biologic mechanisms. Second, with nearly 80,000 IHD hospital admissions over 7 years, our study was well powered to detect statistically significant associations. Moreover, this was one of the few epidemiologic studies that focused on exposure to source-apportioned PM_10_, whereas most available studies were based on source-apportioned PM_2.5_. This allowed us to identify adverse associations of not only sources that primarily generate finer mode PM_10_, but also those that produce coarser mode PM_10_.

Although we provided evidence of the health impacts of several PM_10_ sources in Hong Kong, these findings should be interpreted with caution. Whereas the backward elimination procedure was used to identify a subset of predictors with the most statistically significant relationship with IHD hospitalization, this approach might not guarantee a truly “best” reduced model (Breiman 1996). The importance of PM_10_ sources (e.g., regional combustion, secondary sulfate) excluded from the final multisource model should not be diminished because the statistical elimination procedure does not indicate or account for biological importance. Another limitation of this study was the every-6th-day sampling scheme for the PM_10_ speciation data, resulting in nearly one-third of study days without samples from any stations. Exposure misclassification error might exist; however, our risk estimates were insensitive to alternative interpolation methods (data not shown). Moreover, PM from local emissions (e.g., vehicle exhaust, soil/road dusts) tend to have more error than PM from regional sources (e.g., secondary PM), given their higher spatial heterogeneity (Ito et al. 2004). Such issues of representativeness associated with PM sources may hinder the interpretations of the relative strengths of the observed associations in monitor-based studies of ambient PM pollution.

## Conclusion

We report evidence that PM_10_ from vehicle exhaust, nitrate-rich secondary PM, and sea salt–related PM were significantly associated with elevated IHD hospitalization risks in Hong Kong. This study joins a growing body of literature to report evidence of adverse effects of source-apportioned PM mass, which would help prioritize research on the biologic mechanisms linking PM pollution to cardiac events and guide future monitoring and emission control polices.
